# Assessing long-term effects of gaseous air pollution exposure on mortality in the United States using a variant of difference-in-differences analysis

**DOI:** 10.1038/s41598-024-66951-9

**Published:** 2024-07-13

**Authors:** Yong Yu, Ziqing Tang, Yuqian Huang, Jingjing Zhang, Yixiang Wang, Yunquan Zhang, Qun Wang

**Affiliations:** 1 Center of Health Administration and Development Studies, School of Public Health, Hubei University of Medicine, Shiyan, 442000 China; 2 Institute of Social Development and Health Management, Hubei Province Key Laboratory of Occupational Hazard Identification and Control, School of Public Health, Wuhan University of Science and Technology, Wuhan, 430065 China

**Keywords:** Gaseous pollutant, Causal association, Mortality, Difference-in-differences, Joint effect, Environmental impact, Environmental sciences, Risk factors

## Abstract

Long-term mortality effects of particulate air pollution have been investigated in a causal analytic frame, while causal evidence for associations with gaseous air pollutants remains extensively lacking, especially for carbon monoxide (CO) and sulfur dioxide (SO_2_). In this study, we estimated the causal relationship of long-term exposure to nitrogen dioxide (NO_2_), CO, SO_2_, and ozone (O_3_) with mortality. Utilizing the data from National Morbidity, Mortality, and Air Pollution Study, we applied a variant of difference-in-differences (DID) method with conditional Poisson regression and generalized weighted quantile sum regression (gWQS) to investigate the independent and joint effects. Independent exposures to NO_2_, CO, and SO_2_ were causally associated with increased risks of total, nonaccidental, and cardiovascular mortality, while no evident associations with O_3_ were identified in the entire population. In gWQS analyses, an interquartile range-equivalent increase in mixture exposure was associated with a relative risk of 1.067 (95% confidence interval: 1.010–1.126) for total mortality, 1.067 (1.009–1.128) for nonaccidental mortality, and 1.125 (1.060–1.193) for cardiovascular mortality, where NO_2_ was identified as the most significant contributor to the overall effect. This nationwide DID analysis provided causal evidence for independent and combined effects of NO_2_, CO, SO_2_, and O_3_ on increased mortality risks among the US general population.

## Introduction

Ambient air pollution has posed a significant and persistent threat to global public health over several decades^1^. The Global Burden of Disease (GBD) Project estimated that in 2019, air pollution, as the 4th highest-ranking driver of mortality worldwide, caused approximately 6.67 million premature deaths per year^2^. A large number of population-based studies suggested the associations between prolonged air pollution exposure and heightened risk of total and cause-specific mortality, but these accumulating evidences have been dominated by modeling associations rather than establishing causality^3^. Some scientists advocated for a shift in focus when updating and revising air quality standards, emphasizing the importance of the evidence from causal inference methods rather than relying largely on that of traditional association analysis particularly in cases where the causality of confounding factors was not clear^4^. Estimated effects of exposure from traditional association assessment models often suffered from residual biases introduced by unmeasured confounders^5^, but causal inference models, exemplified by the difference-in-differences (DID) approach, could be recognized as a viable approach to address this issue of residual confounding that frequently bedevils observational investigations^6^.

DID analysis, characterized by its quasi-experimental nature, has gradually gained prominence in the scientific field of environmental epidemiology for estimating causality. Standard DID methods could leverage the assumption of parallel trends and well allow controlling for unobserved confounders, thereby facilitating the identification of causal associations between exposures and outcomes of interest within a counterfactual framework^7^. Building upon this traditional framework, Wang and colleagues developed a variant of DID analysis to quantify the causal effects of particulate air pollutants on mortality through comparing annual fluctuations in mortality and exposure levels across designated geographical units^8^. The revised DID design could not only enhance the capacity to capture the temporal and spatial effects of environmental exposure, but also well account for the potential impacts engendered by socioeconomic factors and unobserved variables^9^.

Several emerging DID studies provided causal evidence linking particulate air pollution with mortality^7,9–11^, while causal effects of gaseous air pollutants remained extensively lacking^12,13^. Moreover, given that individuals were generally exposed to increasingly complex and ever-changing environmental mixtures, there have been surprisingly few studies examining the combined associations of prolonged air pollutants exposure with total and cardiorespiratory mortality^14^. To fill these research gaps, we designed a variant of DID analysis to assess the causal effects of four criteria gaseous air pollutants on mortality based on 108 municipalities in the United States (US) covering the years 1987–2000. The primary objective of this investigation was to examine the individual and joint associations between prolonged exposure to gaseous air pollutants and four mortality endpoints. A secondary objective was to explore the potential effect modifications by age in these causal associations. The investigation into age-related vulnerabilities concerning air pollution exposure could provide a valuable basis for the development of targeted public health interventions, a matter of increasing importance in an aging global population.

## Methods

### Data collection

The principal data for our analysis were sourced from National Morbidity, Mortality, and Air Pollution Study (NMMAPS) database, and the NMMAPS incorporated data from the US National Center for Health Statistics, the 2000 US Census, US Environmental Protection Agency (EPA), and the National Climatic Data Centre^15,16^. The NMMAPS consists of daily time-series data of mortality, air pollution, and weather conditions for 108 municipalities across the continental US over the period 1987–2000 (Fig. S1). Daily counts of deaths from total and nonaccidental causes, as well as cardiovascular disease (CVD) and respiratory disease (RD) were collected from US National Center for Health Statistics and subdivided into three age categories (≤64, 65–74, ≥75 years). Additionally, total population data was sourced from the 2000 U.S. Census. Daily average concentrations of particulate (PM_2.5_ and PM_10_) and gaseous (NO_2_, O_3_, SO_2_, CO) pollutants were averaged across all monitoring sites within each of the metropolitan areas, provided by the US EPA, and daily meteorological data including mean temperatures and relative humidity were derived from the National Climatic Data Centre. A detailed description of the NMMAPS dataset can be found in prior publications^17,18^. In our analytic stage, for each year (1987–2000) and each municipality, daily time-series data of total and cause-specific deaths were aggregated into annual sums, and daily air pollutants and temperatures were summarized as annual averages and seasonal averages or deviations in summers (June to August) and winters (December to February), respectively^19^.

### Statistical analysis

Descriptive characteristics were summarized as means (standard deviations, SDs) for continuous variables or frequencies (proportions) for categorical variables. Spearman rank correlation coefficient (*r*_s_) was used to measure the pairwise correlations among gaseous air pollutants.

#### DID design

A developed variant of the DID approach, a causal modeling method that could control for unmeasured confounders and capture broader temporal trends over the period 1987 to 2000, was utilized to estimate the effect of long-term exposure to each gaseous air pollutant on total and cause-specific mortality^8^. This modified DID analysis was primarily designed in this study through comparing year-to-year (e.g., 1988 vs. 1987, 1989 vs. 1988) changes in annual concentrations of the four gaseous air pollutants (NO_2_, CO, SO_2_, and O_3_) with concurrent changes in total and cause-specific (i.e., nonaccidental, CVD, RD) death number in given 108 municipality units^9^. Under this DID framework, some factors such as socio-economic status, demographic characteristics, and behavioral influences may vary rarely between adjacent years so their confounding effects could be counteracted. However, other time-variant factors that were associated with exposure remained to be adjusted. In accordance to recent DID studies based on aggregated times-series data^9,10,20^, it is hypothesized that such potential confounders might only be seasonal temperatures in our DID analysis on air pollution-mortality associations. The parallel trend assumption is key to causal estimation in DID design, which means that differences in mortality between spatial units in the absence of exposure effects remained stable over time^12^.

### Individual analysis

We used conditional Poisson regression (CPR) model with time-varying exposure at a 1-year scale, to estimate the causal effects of prolonged individual exposure to gaseous pollutants on mortality. CPR model could be a good solution to address the issues of overdispersion and autocorrelation of time series data^20^. We fitted the following DID model to analyze the mortality effects of individual gaseous air pollutants, considering spatial and temporal factors, as well as seasonal variation and population-level characteristics:

$$\text{ln}\left[E\left({Y}_{s,t}\right)\right]={\beta }_{0}+{\beta }_{1}{I}_{s}+{\beta }_{2}{I}_{t}+{\beta }_{3}{Temp}_{sum}+{\beta }_{4}{Temp}_{win}+{\beta }_{5}SD({Temp}_{sum})+{\beta }_{6}SD({Temp}_{win})+{\beta }_{7}{AP}_{s,t}+\text{ln}({Pop}_{s,t})$$

where ${Y}_{s,t}$ represents the number of total and cause-specific deaths in spatial unit *s* (i.e., 108 US municipalities), calendar year *t* (14-y period, 1987–2000). ${AP}_{s,t}$ refers to the annual mean concentrations of air pollutants in spatial unit *s*, calendar year *t*. ${\text{ln}(Pop}_{s,t})$ is an offset term representing the natural logarithm of the population in spatial unit *s*, which controlled for the confounding effect due to city-level population variations. ${I}_{s}$ is a dummy variable for each spatial unit *s*.${I}_{t}$ is a dummy variable for each calendar year *t*. ${\beta }_{0}$ is the fixed intercept term. ${\beta }_{1}$, ${\beta }_{2}$ are regression coefficients of the spatial and temporal effects. ${\beta }_{3},{\beta }_{4},{\beta }_{5},{\beta }_{6}$ are regression coefficients for the effects of mean summer and winter temperatures and their standard deviations, respectively. ${\beta }_{7}$ represents the independent effect of each gaseous pollutant.

The estimated associations were expressed as relative risks (RRs) and their 95% confidence intervals (CIs) per interquartile range (IQR) increase in individual exposure to gaseous air pollutant, and statistical significance was defined as *P* <0.05 (two-tailed). Given the potential departure of linear assumptions in modeling the relationships between independent exposure to four gaseous air pollutants and total and cause-specific mortality, a natural cubic spline (NCS) term of each pollutant with 3 degrees of freedom was incorporated into the CPR model to derive the exposure–response (E-R) association curves^10^. Likelihood ratio test was employed to examine the nonlinearity in associations between gaseous pollutants and mortality via comparing goodness of fit between linear and NCS smoothed models^21^.

Several sensitivity analyses were carried out to testify the robustness of the estimated associations. Firstly, age-stratified analysis was conducted to investigate potentially differential susceptibility to gaseous air pollutants across distinct age groups (≤64, 65–74, and ≥75 years), and meta-regression approach was adopted to examine effect heterogeneity between groups. Secondly, we re-ran the CPR models by extending exposure lag periods, from the current year (lag 0) up to previous 3 years (lag 3) and moving averages of current and previous 1–3 year’s exposure (lag 01, lag 02, and lag 03). Thirdly, bi-, tri-, and quad-pollutant models were fitted to check the influence of co-pollutant adjustments. Finally, since reliable PM_2.5_ monitoring did not begin until around 1999 in the US, we performed co-pollutant analyses by additionally adjusting for PM_10_ in the DID model given the potential confounding effect of particulate air pollution on the mortality effect of gaseous pollutants.

#### gWQS regression analysis

To measure the overall joint effect of NO_2_, CO, SO_2_, and O_3_ along with their relative contributions to the targeted mortality outcomes, we introduced the generalized weighted quantile sum regression (gWQS) by constructing a gWQS index to facilitate mixture-exposure analysis^6^. Pollutant exposures were classified into quartiles separately and combined into a gWQS index by determining weights according to their relevance to the overall associations with the outcome, which could thus reduce dimensionality and avoid covariance issues^22^. In this study, we incorporated the gWQS approach into main CPR analysis to estimate the combined effect and relative contributions of individual gaseous pollutants to the overall mortality effect. The formula of gWQS model was specified as follows:

$$\text{ln}\left[E\left({Y}_{s,t}\right)\right]={\beta }_{0}+{\beta }_{1}{I}_{s}+{\beta }_{2}{I}_{t}+{\beta }_{3}{Temp}_{sum}+{\beta }_{4}{Temp}_{win}+{\beta }_{5}SD({Temp}_{sum})+{\beta }_{6}SD({Temp}_{win})+{\beta }_{7}{AP}_{s,t}+{\beta }_{8}\sum_{i=1}^{4}{w}_{i}{q}_{i}+\text{ln}({Pop}_{s,t}){|}_{b}$$

where ${\beta }_{8}$ represents the regression coefficient of the gWQS index; ${q}_{i}$ (0, 1, 2, 3) denotes the quantile (1st, 2nd, 3rd, or 4th) of component $i$. ${w}_{i}$ was the estimated weight (0–1) of the ${i}^{th}$ component. *b* denotes the ${b}^{th}$ bootstrap step. $\sum_{i=1}^{4}{w}_{i}{q}_{i}$ refers to the weighted index for the set of four targeted air contaminants. The final weights for air pollutants were obtained by averaging all the weights with significant coefficient of overall mixture effect^23^. The sum of all weights is equal to 1, with larger weights indicating greater contributions in the overall effects. We randomly split the data into training set (40%) for estimating empirical weights and validation set (60%) for deriving statistical significance. A total of 1000 bootstrap steps were performed in the training set to increase the sensitivity in detecting significant predictors and obtain stable weights. We finally set the direction of the regression coefficient effect to be positive based on the results of single-pollutant DID model^24^. The combined effect was presented as RR (95% CI) of total and cause-specific mortality per IQR-equivalent increase of exposure in mixture pollutants.

All statistical analyses were conducted using the R version 4.3.1 (R Foundation for Statistical Computing, Vienna, Austria), with the package “gnm” (version 1.1-2) for the CPR model analysis, “mvmeta” (version 1.0.3) for comparing effect differences between subgroups, “splines” (version 4.3.1) for smoothing NCS term, and “gWQS” (version 3.0.4) for assessing the joint associations.

## Results

Table 1 describes the summary statistics of deaths, gaseous air pollutants, and climate variables in the 108 US municipalities from 1987 to 2000. We observed 10,794,553 total and 10,395,583 nonaccidental deaths, including 4,546,977 and 894,157 deaths from cardiovascular and respiratory diseases, respectively. Annual mean (SD) concentrations of gaseous air pollutants were 20.88 (6.65) ppb for NO_2_, 5.43 (3.59) ppb for SO_2_, 26.45 (5.28) ppb for O_3_, and 0.94 (0.37) ppm for CO, respectively (Fig. S1). NO_2_, CO, and SO_2_ exhibited low-to-moderate correlations (0.25 < *r*_s_ < 0.52) with each other, but were inversely and lowly correlated with O_3_ (− 0.25 < *r*_s_ < − 35, Fig. S2).

**Table 1:** Summary statistics of annual deaths, air pollution levels, and meteorological factors in 108 US municipalities over the period 1987–2000.

Characteristic	Total (%)	Mean (SD)	Min	P_25_	Median	P_75_	Max	IQR
Total death	10,794,553 (100)	7139 (9665)	1278	2628	4434	7542	79,432	4914
Nonaccidental	10,395,583 (96.3)	6875 (9357)	748	2506	4270	7253	77,044	4747
CVD	4,546,977 (42.1)	3007 (4401)	177	1181	1828	3041	36,276	1860
RD	894,157 (8.3)	591 (779)	37	191	390	680	6188	489
Age ≤64	3,019,463 (28.0)	1997 (2968)	378	767	1162	2053	26,417	1286
Age 65–74	2,393,268 (22.2)	1583 (1919)	373	702	1038	1624	15,925	922
Age ≥75	5,381,822 (50.0)	3559 (4856)	470	1192	2171	3782	37,426	2590
Air pollution								
NO_2_ (ppb)	–	20.88 (6.65)	3.98	16.86	20.35	24.84	45.40	7.98
CO (ppm)	–	0.94 (0.37)	0	0.70	0.88	1.10	2.46	0.40
SO_2_ (ppb)	–	5.43 (3.59)	0.03	2.52	4.64	7.38	19.97	4.86
O_3_ (ppb)	–	26.45 (5.28)	2.21	23.07	26.45	29.64	43.44	6.57
PM_10_ (μg/m^3^)	–	29.75 (6.31)	14.56	24.62	29.71	34.27	44.28	9.65
Seasonal temperature								
Mean winter temperature (℃)	–	5.63 (6.61)	10.93	0.15	5.29	11.10	24.17	10.95
Mean summer temperature (℃)	–	24.96 (3.63)	13.86	22.62	25.35	27.12	37.70	4.50
SD of winter temperature (℃)	–	5.16 (1.53)	1.10	4.30	5.18	6.22	8.74	1.92
SD of summer temperature (℃)	–	3.08 (0.86)	0.52	2.44	3.18	3.64	5.53	1.20

The sum of percentages from multiple subgroups may not equal 100% exactly due to rounding-off numbers.

*SD* standard deviation, *Min* minimum value, *Max* maximum value, *IQR* interquartile range, *NO*_*2*_ nitrogen dioxide, *CO* carbon monoxide, *SO*_*2*_ sulfur dioxide, *O*_*3*_ ozone, *PM*_*10*_ particulate matter 10 µm or less in diameter, *CVD* cardiovascular disease, *RD* respiratory disease, *ppb* part per billion, *ppm* part per million.

**Figure 1:**
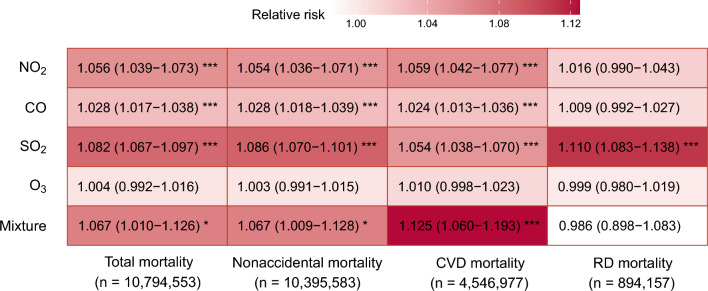
Risk estimates (with 95% CIs) of total and cause-specific mortality associated with per IQR increase in O_3_, SO_2_, NO_2_, CO, and an IQR-equivalent increase in mixture exposure. *NO*_*2*_ nitrogen dioxide, *CO* carbon monoxide, *SO*_*2*_ sulfur dioxide, *O*_*3*_ ozone, *CVD* cardiovascular disease, *RD* respiratory disease, *CI* confidence interval, *IQR* interquartile range.

**Figure 2:**
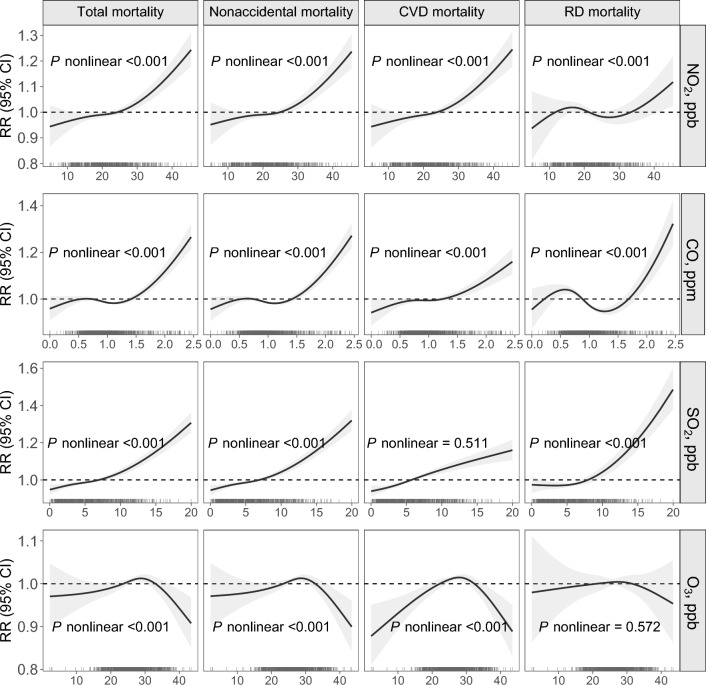
Estimated exposure–response curves for the associations of gaseous pollutant concentrations with total, nonaccidental, cardiovascular, and respiratory mortality. The solid black line represents the effect estimates and the shaded area represents its 95% confidence interval. *NO*_*2*_ nitrogen dioxide, *CO* carbon monoxide, *SO*_*2*_ sulfur dioxide, *O*_*3*_ ozone, *CVD* cardiovascular disease, *RD* respiratory disease, *RR* relative risk, *CI* confidence interval, *ppb* part per billion, *ppm* part per million.

Figure 1 estimates the associations between mortality endpoints and independent and joint exposure to four criteria gaseous air pollutants. Single exposures to NO_2_, CO, and SO_2_ were consistently associated with increased total, nonaccidental, and CVD mortality, while no evidence for O_3_-mortality associations was seen in the entire population. For instance, the RR estimates of total mortality were 1.056 (95% CI: 1.039–1.073), 1.082 (1.067–1.097), 1.004 (0.992–1.016), and 1.028 (1.017–1.038) for each IQR increase in NO_2_, SO_2_, O_3_, and CO exposure, respectively. Only SO_2_ was found to be significantly associated with elevated RD mortality, with an excess risk of 11.0% (8.3%–13.8%) for each IQR rise in exposure. These findings were largely robust to sensitivity analyses via using various lag periods and fitting co-pollutant models (Table 2, Tables S1, S2). Traditional and DID analyses based on NMMAPS provided consistent associations between gaseous pollution and mortality, and the association magnitudes were found to be comparable in both estimates. Overall, approximately J-shaped curves with steeper slopes under higher exposure conditions were observed for the exposure–response relationships of mortality risks with NO_2_, CO, and SO_2_ exposure, while O_3_-associated risk curves tended to be inverted U-shaped (Fig. 2).

**Table 2:** Risk estimates (with 95% CIs) of total and cause-specific mortality associated with per IQR increase in individual and mixture exposure to gaseous air pollutants across different lag years.

Pollutant	Lag (years)	RR (95% CI), per IQR increase			
		Total mortality	Nonaccidental mortality	CVD mortality	RD mortality
NO_2_ (ppb)	1	1.047 (1.029–1.064)	1.043 (1.026–1.061)	1.046 (1.028–1.065)	1.004 (0.977–1.032)
	2	1.052 (1.034–1.069)	1.050 (1.032–1.068)	1.052 (1.033–1.071)	1.016 (0.988–1.046)
	3	1.011 (0.993–1.029)	1.011 (0.993–1.030)	1.017 (0.998–1.037)	0.968 (0.938–0.999)
	01	1.061 (1.041–1.082)	1.057 (1.036–1.078)	1.066 (1.044–1.088)	1.010 (0.977–1.043)
	02	1.062 (1.037–1.087)	1.058 (1.033–1.084)	1.070 (1.043–1.097)	1.007 (0.965–1.051)
	03	1.055 (1.027–1.084)	1.052 (1.023–1.081)	1.062 (1.032–1.093)	0.987 (0.939–1.037)
CO (ppm)	1	1.016 (1.005–1.027)	1.016 (1.005–1.027)	1.016 (1.004–1.028)	0.994 (0.976–1.013)
	2	1.008 (0.998–1.019)	1.008 (0.997–1.019)	1.012 (1.000–1.024)	0.986 (0.967–1.006)
	3	0.997 (0.987–1.008)	0.997 (0.987–1.008)	1.005 (0.994–1.017)	0.983 (0.963–1.003)
	01	1.028 (1.016–1.039)	1.028 (1.016–1.040)	1.024 (1.012–1.037)	0.999 (0.979–1.020)
	02	1.029 (1.016–1.042)	1.029 (1.016–1.042)	1.024 (1.010–1.038)	0.989 (0.966–1.013)
	03	1.029 (1.015–1.044)	1.029 (1.014–1.044)	1.025 (1.010–1.041)	0.987 (0.961–1.015)
SO_2_ (ppb)	1	1.075 (1.060–1.091)	1.078 (1.063–1.094)	1.052 (1.036–1.069)	1.098 (1.070–1.127)
	2	1.070 (1.055–1.085)	1.073 (1.058–1.088)	1.053 (1.037–1.069)	1.093 (1.064–1.122)
	3	1.062 (1.046–1.078)	1.062 (1.046–1.079)	1.051 (1.033–1.068)	1.057 (1.025–1.091)
	01	1.092 (1.076–1.109)	1.096 (1.079–1.113)	1.061 (1.043–1.079)	1.121 (1.091–1.153)
	02	1.096 (1.078–1.114)	1.100 (1.082–1.119)	1.062 (1.043–1.082)	1.134 (1.098–1.170)
	03	1.094 (1.074–1.114)	1.099 (1.079–1.119)	1.067 (1.046–1.089)	1.141 (1.098–1.184)
O_3_ (ppb)	1	1.005 (0.993–1.017)	1.004 (0.992–1.016)	1.014 (1.001–1.027)	0.994 (0.975–1.014)
	2	0.996 (0.984–1.007)	0.995 (0.983–1.006)	1.006 (0.994–1.019)	0.972 (0.953–0.992)
	3	0.998 (0.987–1.009)	0.998 (0.987–1.009)	1.004 (0.992–1.016)	0.987 (0.967–1.007)
	01	1.005 (0.991–1.019)	1.004 (0.990–1.018)	1.017 (1.002–1.033)	0.997 (0.974–1.020)
	02	1.002 (0.986–1.018)	1.000 (0.984–1.016)	1.015 (0.999–1.033)	0.982 (0.956–1.009)
	03	1.001 (0.984–1.018)	0.999 (0.982–1.016)	1.014 (0.995–1.033)	0.968 (0.939–0.998)
Mixture	1	1.036 (0.982–1.093)	1.025 (0.972–1.081)	1.094 (1.037–1.154)	0.966 (0.874–1.067)
	2	0.993 (0.936–1.053)	0.993 (0.936–1.052)	1.051 (0.988–1.118)	0.903 (0.844–0.967)
	3	1.036 (0.983–1.092)	1.038 (0.983–1.096)	1.077 (1.023–1.133)	0.912 (0.815–1.021)
	01	1.038 (0.967–1.114)	1.035 (0.964–1.111)	1.096 (1.034–1.162)	0.950 (0.836–1.078)
	02	1.112 (1.043–1.185)	1.114 (1.043–1.190)	1.173 (1.096–1.256)	1.118 (1.002–1.248)
	03	1.026 (0.939–1.121)	1.018 (0.931–1.112)	1.089 (0.992–1.195)	0.920 (0.804–1.052)

*RR* relative risk, *CI* confidence interval, *NO*_*2*_ nitrogen dioxide, *CO* carbon monoxide, *SO*_*2*_ sulfur dioxide, *O*_*3*_ ozone, *CVD* cardiovascular disease, *RD* respiratory disease, *IQR* interquartile range, *ppb* part per billion, *ppm* part per million.

**Figure 3:**
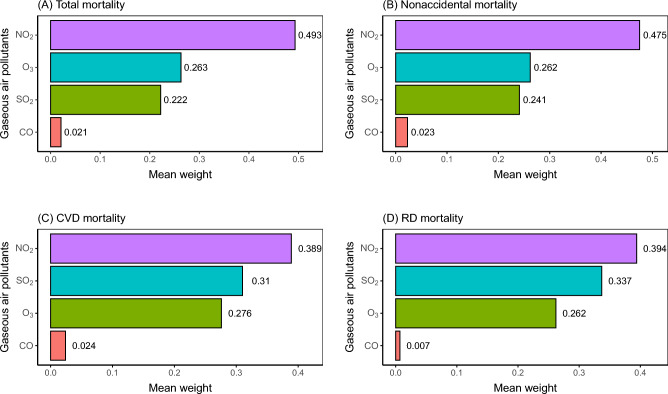
gWQS-derived mean weights of each gaseous air pollutants in the joint effects of mixture exposure on total and cause-specific mortality. *NO*_*2*_ nitrogen dioxide, *CO* carbon monoxide, *SO*_*2*_ sulfur dioxide, *O*_*3*_ ozone, *CVD* cardiovascular disease, *RD* respiratory disease.

In the multi-pollutant analysis via gWQS regression, one IQR-equivalent increase in mixture exposure was associated with an estimated RR of 1.067 (1.010–1.126) for total mortality, 1.067 (1.009–1.128) for nonaccidental mortality, 1.125 (1.060–1.193) for CVD mortality, and 0.986 (0.898–1.083) for RD mortality, respectively (Fig. 1). Figure 3 displays relative contributions (weights) of each gaseous air pollutant in the overall effects on mortality estimated by gWQS models. The combined mortality effects of exposure to four gaseous pollutants were mostly contributed by NO_2_ but least contributed by CO. Specifically, NO_2_ was responsible for 38.9%–49.3% of the increased mortality risks related to overall mixture exposure, while CO accounted for only 0.7%–2.4% of the joint associations with total and cause-specific mortality. It is noteworthy that the relative importance measured by gWQS weights did not match the sequence of effect sizes estimated by the single-pollutant DID models.

**Figure 4:**
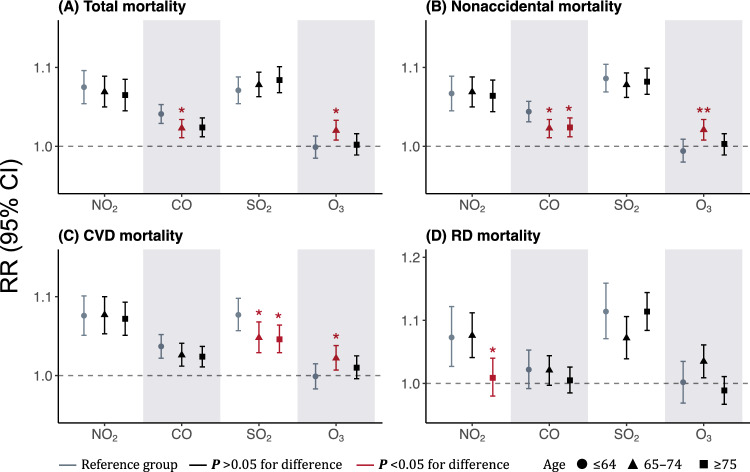
Age-stratified associations between total and cause-specific mortality and gaseous air pollution exposure. *NO*_*2*_ nitrogen dioxide, *CO* carbon monoxide, *SO*_*2*_ sulfur dioxide, *O*_*3*_ ozone, *CVD* cardiovascular disease, *RD* respiratory disease, *RR* relative risk, *CI* confidence interval; *P <0.05, **P <0.01.

Figure 4 illustrates age-stratified risk estimates for mortality associated with individual exposure to gaseous pollutants. A mixed pattern was detected for age modification in pollutants-mortality associations. Our stratified analysis revealed evidently higher CO-related risks in younger group aged ≤64 years, with estimated RRs of 1.041 (1.029–1.053) for total mortality, 1.044 (1.031–1.057) for nonaccidental mortality, 1.037 (1.022–1.052) for CVD mortality, and 1.022 (0.992–1.053) for RD mortality associated with one IQR increase in CO exposure, respectively (Table S3). Despite no O_3_-related excess risks in the entire population, O_3_-mortality associations were found to be more pronounced in individuals aged 65–74 years. For total mortality, per IQR increase in O_3_ was associated with a risk of 1.020 (1.008–1.033) in those aged 65–74 years, 0.999 (0.985–1.013) in younger group (≤64 years) and 1.002 (0.989–1.016) in older group (≥75 years), respectively. In terms of NO_2_ and SO_2_, no evidence for age modification was identified in this analysis (*P* >0.05 for heterogeneity).

## Discussion

In the context of decades of rapid industrial growth in the United States, coupled with challenges within environmental legislation, air pollution and mortality have been on a parallel upward trend over the 14-year period 1987–2000. This nationwide DID study provided compelling evidence for elevated risks for total, nonaccidental, and CVD mortality in the US population causally associated with independent and combined exposures to NO_2_, CO, SO_2_, and O_3_. Mixture effects associated with co-exposure to four gaseous pollutants were predominantly attributed to NO_2_. Our study identified approximately J-shaped curves for mortality outcomes in association with NO_2_, CO, and SO_2_, while O_3_-associated risk curves tended to be inverted U-shaped. Age-stratified analysis revealed mixed patterns of effect modification in exposure-mortality associations. This present study is essential in furnishing supplementary causal evidence derived from observational data, thereby substantiating the revision of air quality guidelines.

Our adapted DID analysis demonstrated robustly positive associations of long-term NO_2_ exposure with mortality of US population, echoing with a growing body of epidemiological studies relying on several causal modelling frameworks^3^. Causal evidence for excess mortality risks due to long-term NO_2_ exposure were reported in an Australian DID study of 2193 statistical areas level-2^12^, instrumental variable (IV)-based analysis of 7.3 million deaths in 135 US cities^25^, and US national Medicare cohort analyses based on inverse probability weighting (IPW)^26^ and generalized propensity score (GPS)^27^. Similarly, in a pooled analysis from 8 European cohorts^28^, consistently positive NO_2_-mortality nexuses were seen through parallel Cox modelling strategies weighted by IPW and adjusted for GPS, wherein both approaches estimated largely comparable effect sizes. Also, our study generally supported the causal evidence for elevated mortality risk associated with long-term exposure to SO_2_ and CO. While SO_2_-associated risks were reported in another two DID analyses in China^13^ and Germany^29^, such causal evidence for CO in the general population remains extensively sparse to date. Collectively, mortality relationships with SO_2_ and CO were between suggestive and causal, as summarized by US Environmental Protection Agency^30,31^.

For O_3_-mortality associations, given the heterogeneous findings from population-based cohorts in the USA and Europe^32^, we believed that the evidence suggested "likely to be causal" for respiratory effects and “suggestive of a causal relationship” for cardiovascular effects^33^. Inconsistent with one Korean IV analysis^34^ and two US cohort studies (using GPS and IPW)^26,27^, our DID analysis did not identify causally positive relationship between annual O_3_ exposure and total and cardiopulmonary mortality. These null associations were also reported in a prior review of 20 traditional cohort analyses focusing on annual O_3_ metric^35^. Overall, current cohort evidence on O_3_-mortality association was largely mixed and exhibited considerable regional heterogeneity^36^. On the whole, national cohorts in North America^37–39^ and China^40–42^ linked elevated mortality risks (e.g., all-cause, CVD, and RD) with annual average or peak-season O_3_ exposure. This was in marked contrast to the large volume of epidemiological cohort evidence from South Korea^43^ and European countries such as Denmark^44^, France^45^, and England^46^, which mostly reported null or protective effects on long-term survival. Several factors such as demographic characteristics, control of potential confounders, air pollution levels, different exposure metrics and analytic methodology might be responsible for regional variations in observed mortality effects^44,47^.

As suggested in our mixture exposure analysis, NO_2_ constituted the most prominent contributor to the joint effects of four criteria gaseous air pollutants on excess risk of mortality. In a 22-year cohort study in Northern China, PM_2,5_ was reported as the principal contributor to the conjoined cardiopulmonary mortality effects of exposure mixture (PM_10_, PM_2.5_, SO_2_, and NO_2_)^14^. Generally consistent evidence was seen in two investigations^48,49^ from developed countries (Canada and the US), suggesting PM_2.5_-dominated mortality effects under co-existing exposure scenario with NO_2_. This further could attest to a discernible corollary that the detrimental effects of NO_2_ could be possibly masked to a considerable extent by particulate matter, particularly PM_2.5_^50^. It has been evidenced that the presence of PM_2.5_ may exert an attenuating influence upon the fatality effects of NO_2_, given that both PM_2.5_ and NO_2_ are atmospheric pollutants emanating from common sources, such as vehicular emissions and industrial activities^51^. Additonally, the greatest association estimates of mixture pollutant exposure were seen at lag 02 year (Table 2), suggesting a cumulative effect of gaseous pollutants on mortality risk. In accordance with prior DID analyses^9,52^, the risk effects for multi-year lags were marginally larger and more stable than those for single-year lags, which well underscored the importance of considering accumulated exposure in environmental health-risk assessments. Currently available investigations pertaining to the co-exposure of air pollutants remain relatively lacking, and the complexity arising from variations in geographical locations, analytic methodologies, and combinations of pollutants may introduce great challenges in achieving comparative evidence on mixed exposures. Therefore, national and global initiatives are pressingly needed to illuminate the intricate interplay of combined exposure to multiple atmospheric pollutants and its multifaceted implications for human health.

There have been mixed findings on effect modification by age concerning the associations of gaseous pollutants with mortality. In this study, individuals aged ≤64 years showed higher CO-associated risks of total and cardiorespiratory mortality, echoing with two multi-city time-series studies in China^53,54^. Additionally, we observed higher O_3_-associated mortality risks among individuals aged 65–74 years. In two Chinese national cohort studies, more pronounced O_3_-mortality associations were reported in older adults aged ≥65 years^40,42^. By comparison, evidence from mega cohorts suggested no age difference among older people (65 years and older) in US^55^ and higher risk in younger (age ≤60 years) group in Rome^56^. Age disparities in O_3_-mortality associations could be possibly related to geographical and demographic variations between studies, as well as differential population exposure levels and time-activity patterns^39,44^. In accordance with three preceding investigations from developed countries (i.e., US and Canada)^37,57^ and China^58^, we found no evidence of effect modification by age on association between SO_2_/NO_2_ and mortality. Notably, both pollutants demonstrated a positive linkage with total and cardiopulmonary mortality across diverse age strata. As such, fostering a synergistic approach to govern and mitigate the mortality risks of these air pollutants should become an indispensable imperative, in order to achieve a state of collective health benefits in the general population.

A major feature of this study is the DID causal analysis which effectively controls for unmeasured confounders in the causal links between gaseous air pollutants and total as well as cause-specific mortality. Expanding upon the conventional DID approach, the DID “variant” well controls for additionally potential confounders (e.g., seasonal factors) that fluctuate across locations and over time, thereby mitigating potential biases in estimating exposure effects and capturing well long-term trends in gaseous pollution-morality associations. Furthermore, the application of the gWQS model in our study provides an enhanced exploration on the joint mortality effects of combined exposure to gaseous pollutants and its individual relative contributions. Nevertheless, this study has several limitations that should be acknowledged. First, although the variant DID method counteracts the effects of temporally stable individual and behavioral factors (including unmeasured ones), it remains plausible that there exist additional latent confounding factors, including socioeconomic status, health behaviors (e.g., smoking), and changes in residential characteristics, which could vary across location and time^9^. Additionally, DID model has the potential power for causal inference and advantages over traditional analytical approaches by reducing confounding, but established causality on the basis of data analysis still need to be validated physiologically by high-quality human and animal experiments. Second, exposure misclassifications may arise from our exclusive utilization of population-level air pollution data rather than incorporating more granular data sources such as individual-level measurements^11^. Third, some missing records of daily time series air pollution data in NMMAPS database, may introduce some uncertainty into the effect estimates but its impact on the association is deemed to be relatively inconspicuous^59^. Forth, one of the fundamental assumptions of the gWQS method is unidirectional exposure effect, encompassing either a positive or negative trend. However, this assumption may potentially lead to issues such as non-convergence of the model or biased estimation^60^. Fifth, the NMMAPS database comprised predominantly outdated monitoring data, and therefore the results may not be representative of current exposures and pollution sources.

## Conclusions

In summary, our DID analysis provided compelling evidence for elevated risks of total, nonaccidental, and CVD mortality causally associated with both combined and independent exposure to gaseous air pollutants in US population. Age-specific analyses produced very mixed results of effect modification regarding long-term effects of gaseous pollutants on mortality, emphasizing the necessity for further investigation of age-related vulnerabilities with the aim of pinpointing specific susceptible populations at higher risks and ultimately contributed to more effective public health. Our study added national evidence of causality and highlighted that targeted policies should be formulated to strengthen the response to the health threats of gaseous pollutant exposure.

### Supplementary Information

Supplementary Information.

## Data Availability

The datasets used and/or analysed during the current study available from the corresponding author on reasonable request.
