# Supplementary material for: Assessing long-term effects of gaseous air pollution exposure on mortality in the United States using a variant of difference-in-differences analysis
Source: Sci Rep. 2024 Jul 13;14:16220. doi: 10.1038/s41598-024-66951-9 (PMC11246484; doi:10.1038/s41598-024-66951-9)
Supplement: Supplementary file 1 — Supplementary Information. [file 41598_2024_66951_MOESM1_ESM.pdf]

# Supplementary materials

## Table of Contents


**Section 1 Supplementary data & methods**

1.1 Causal analytical frame

1.2 Conditional Poisson regression

1.3 Generalized weighted quantile regression

**Section 2 Supplementary tables & figures**

**Table S1** Risks estimates (with 95% CIs) of total and cause-specific mortality associated with each IQR increase in $NO_2$, $SO_2$, $O_3$, and CO using single-, bi-, tri- and quad-pollutant models.

**Table S2** Risks of total and cause-specific mortality associated with each IQR increase in individual gaseous pollutant or IQR-equivalent rise in mixture exposure, estimated by co-pollutant analyses by additionally adjusting for $PM_{10}$.

**Table S3** Age-stratified associations between long-term exposure to gaseous air pollutants and mortality outcomes.

**Fig. S1** Annual average concentrations of specific gaseous pollutants ($NO_2$, CO, $SO_2$, $O_3$) in 108 US cities spanning the period 1987−2000.

**Fig. S2** Spearman correlation coefficients between air pollutants in 108 US cities for the period 1987−2000.

# Section 1 Supplementary data & methods

## 1.1 Causal analytical frame

Causality is a generic relationship between an effect and a cause that produces it. This relationship should be precisely established in the context of physiological mechanisms, rather than solely relying on statistical analysis. In the realm of environmental epidemiology, the predominant approach has traditionally involved modeling associations, typically by regressing observed outcomes against linear or nonlinear functions of observed covariates. However, describing associations between exposures and outcomes generally does not suffice, and an assessment of causal effects is needed[1]. Causal effects refer to the effects that would be seen under experimental changes of exposures. By causal inference we mean the process of inferring causal effects from data[2]. Hence, a causal inference framework should be considered when analyzing the impact of environmental exposures on health outcomes. Such a causal inference framework is a set of statistical frameworks for analyzing causal relationships between exposures and outcomes based on counterfactual estimation of observational data. Three analytical frameworks have been proposed, namely the counterfactual framework, the latent outcome model and the structural causal model.

## 1.2 Conditional Poisson regression

Conditional Poisson regression serves as a pivotal tool used to model count data and contingency tables. Poisson regression assumes the response variable Y has a Poisson distribution, and assumes the logarithm of its expected value can be modeled by a linear combination of unknown parameters. Conditional Poisson models emerge as an invaluable counterpart to unconditional ones, adept at accommodating overdispersion and autocorrelation of time-series data[3].

## 1.3 Generalized weighted quantile regression

Generalized weighted quantile regression (gWQS) is a convenient tool for solving the problem of high dimensionality and high correlation between multiple pollutants, especially homologous pollutants[4]. The method produces results with good interpretability and effectively identifies high-risk factors. The principle of the gWQS regression model is to combine the effects of multiple factors into a single index by means of interquartile spacing and weighting, and then to perform regression analyses. The assigned weights reflecting the magnitude of influence exerted by different factors on outcomes[5].

# References


1 Dominici, F. & Zigler, C. Best Practices for Gauging Evidence of Causality in Air Pollution Epidemiology. *Am J Epidemiol* **186**, 1303-1309, doi:10.1093/aje/kwx307 (2017).

2 Carone, M., Dominici, F. & Sheppard, L. In Pursuit of Evidence in Air Pollution Epidemiology: The Role of Causally Driven Data Science. *Epidemiology* **31**, 1-6, doi:10.1097/ede.0000000000001090 (2020).

3 Renzi, M. *et al.* Long-Term $PM_{10}$ Exposure and Cause-Specific Mortality in the Latium Region (Italy): A Difference-in-Differences Approach. *Environ Health Perspect* **127**, 67004, doi:10.1289/ehp3759 (2019).

4 Traini, E. *et al.* A Multipollutant Approach to Estimating Causal Effects of Air Pollution Mixtures on Overall Mortality in a Large, Prospective Cohort. *Epidemiology* **33**, 514-522, doi:10.1097/ede.0000000000001492 (2022).

5 Hærvig, K. K. *et al.* Maternal Exposure to Per- and Polyfluoroalkyl Substances (PFAS) and Male Reproductive Function in Young Adulthood: Combined Exposure to Seven PFAS. *Environ Health Perspect* **130**, 107001, doi:10.1289/ehp10285 (2022).

## Section 2 Supplementary tables & figures

**Table S1** Risks estimates (with 95% CIs) of total and cause-specific mortality associated with each IQR increase in $NO_2$, $SO_2$, $O_3$, and CO using single-, bi-, tri- and quad-pollutant models.

| Pollutant | Model | RR (95% CI), per IQR increase | | | |
|---|---|---|---|---|---|
| | | **Total mortality** | **Nonaccidental mortality** | **CVD mortality** | **RD mortality** |
| **$NO_2$** | **Single-pollutant** | 1.056 (1.039–1.073) | 1.054 (1.036–1.071) | 1.059 (1.042–1.077) | 1.016 (0.990–1.043) |
| | **Bi-pollutant** | | | | |
| | $+SO_2$ | 1.064 (1.048–1.080) | 1.061 (1.045–1.077) | 1.067 (1.049–1.085) | 1.025 (0.998–1.052) |
| | +CO | 1.052 (1.035–1.069) | 1.048 (1.031–1.066) | 1.058 (1.040–1.077) | 1.021 (0.993–1.049) |
| | $+O_3$ | 1.059 (1.042–1.077) | 1.057 (1.039–1.075) | 1.065 (1.046–1.084) | 1.021 (0.993–1.050) |
| | **Tri–pollutant** | | | | |
| | $+O_3+SO_2$ | 1.066 (1.049–1.083) | 1.063 (1.046–1.080) | 1.071 (1.053–1.090) | 1.028 (1.000–1.056) |
| | $+O_3$+CO | 1.056 (1.038–1.074) | 1.053 (1.034–1.072) | 1.064 (1.045–1.084) | 1.030 (1.000–1.061) |
| | $+SO_2$+CO | 1.059 (1.042–1.076) | 1.055 (1.038–1.073) | 1.063 (1.045–1.082) | 1.029 (1.001–1.058) |
| | **Quad-pollutant** | | | | |
| | $+CO+SO_2+O_3$ | 1.061 (1.043–1.079) | 1.058 (1.040–1.077) | 1.067 (1.047–1.087) | 1.035 (1.005–1.066) |
| **CO** | **Single-pollutant** | 1.028 (1.017–1.038) | 1.028 (1.018–1.039) | 1.024 (1.013–1.036) | 1.009 (0.992–1.027) |
| | **Bi-pollutant** | | | | |
| | $+NO_2$ | 1.029 (1.016–1.043) | 1.031 (1.017–1.044) | 1.020 (1.006–1.035) | 1.012 (0.989–1.036) |
| | $+SO_2$ | 1.028 (1.016–1.040) | 1.028 (1.015–1.040) | 1.027 (1.014–1.040) | 0.993 (0.973–1.014) |
| | $+O_3$ | 1.029 (1.018–1.040) | 1.029 (1.018–1.041) | 1.025 (1.013–1.037) | 1.007 (0.988–1.026) |
| | **Tri-pollutant** | | | | |
| | $+O_3+SO_2$ | 1.026 (1.013–1.039) | 1.025 (1.012–1.038) | 1.026 (1.012–1.040) | 0.989 (0.967–1.011) |
| | $+O_3+NO_2$ | 1.028 (1.014–1.042) | 1.029 (1.015–1.044) | 1.018 (1.003–1.033) | 1.007 (0.983–1.032) |
| | $+SO_2+NO_2$ | 1.018 (1.004–1.032) | 1.019 (1.004–1.033) | 1.014 (0.999–1.029) | 0.990 (0.965–1.015) |
| | **Quad-pollutant** | | | | |
| | $+NO_2+SO_2+O_3$ | 1.014 (1.000–1.029) | 1.015 (1.000–1.030) | 1.011 (0.995–1.027) | 0.984 (0.959–1.011) |

| | | | | | |
|---|---|---|---|---|---|
| **$SO_2$** | **Single-pollutant** | 1.082 (1.067–1.097) | 1.086 (1.070–1.101) | 1.054 (1.038–1.070) | 1.110 (1.083–1.138) |
| | **Bi-pollutant** | | | | |
| | +$NO_2$ | 1.079 (1.062–1.096) | 1.083 (1.066–1.100) | 1.049 (1.031–1.067) | 1.107 (1.076–1.139) |
| | +CO | 1.072 (1.056–1.088) | 1.075 (1.059–1.092) | 1.042 (1.026–1.060) | 1.111 (1.082–1.141) |
| | +$O_3$ | 1.093 (1.077–1.110) | 1.097 (1.080–1.114) | 1.061 (1.044–1.079) | 1.124 (1.095–1.154) |
| | **Tri-pollutant** | | | | |
| | +$NO_2$+CO | 1.074 (1.056–1.093) | 1.078 (1.060–1.096) | 1.045 (1.026–1.064) | 1.114 (1.081–1.149) |
| | +$NO_2$+$O_3$ | 1.086 (1.068–1.105) | 1.090 (1.071–1.109) | 1.054 (1.034–1.074) | 1.114 (1.081–1.149) |
| | +CO+$O_3$ | 1.084 (1.067–1.102) | 1.088 (1.070–1.106) | 1.052 (1.033–1.071) | 1.127 (1.095–1.161) |
| | **Quad-pollutant** | | | | |
| | +CO+$NO_2$+$O_3$ | 1.083 (1.063–1.103) | 1.087 (1.067–1.107) | 1.053 (1.032–1.075) | 1.123 (1.086–1.162) |
| **$O_3$** | **Single-pollutant** | 1.004 (0.992–1.016) | 1.003 (0.991–1.015) | 1.010 (0.998–1.023) | 0.999 (0.980–1.019) |
| | **Bi-pollutant** | | | | |
| | +$NO_2$ | 0.978 (0.961–0.996) | 0.977 (0.960–0.995) | 0.982 (0.964–1.001) | 0.978 (0.950–1.008) |
| | +CO | 1.003 (0.990–1.015) | 1.002 (0.990–1.015) | 1.014 (1.000–1.027) | 0.996 (0.975–1.017) |
| | +$SO_2$ | 1.007 (0.994–1.021) | 1.007 (0.993–1.021) | 1.011 (0.995–1.026) | 1.000 (0.976–1.024) |
| | **Tri-pollutant** | | | | |
| | +$NO_2$+$SO_2$ | 0.991 (0.974–1.008) | 0.990 (0.973–1.008) | 0.994 (0.976–1.013) | 0.990 (0.960–1.021) |
| | +$NO_2$+CO | 0.979 (0.962–0.997) | 0.978 (0.960–0.996) | 0.988 (0.969–1.006) | 0.968 (0.938–0.999) |
| | +$SO_2$+CO | 1.011 (0.995–1.026) | 1.010 (0.994–1.026) | 1.017 (1.000–1.035) | 0.996 (0.970–1.023) |
| | **Quad-pollutant** | | | | |
| | +$NO_2$+CO+$SO_2$ | 0.995 (0.977–1.014) | 0.995 (0.976–1.014) | 1.003 (0.983–1.024) | 0.986 (0.954–1.020) |

Abbreviations: RR, relative risk; CI, confidence interval; $NO_2$, nitrogen dioxide; CO, carbon monoxide; $SO_2$, sulfur dioxide; $O_3$, ozone; CVD, cardiovascular disease; RD, respiratory disease; IQR, interquartile range.

**Table S2** Risks of total and cause-specific mortality associated with each IQR increase in individual gaseous pollutant or IQR-equivalent rise in mixture exposure, estimated by co-pollutant analyses by additionally adjusting for $PM_{10}$.

| Pollutant | RR (95% CI), per IQR or IQR-equivalent increase | | | |
|---|---|---|---|---|
| | **Total mortality** | **Nonaccidental mortality** | **CVD mortality** | **RD mortality** |
| **$NO_2$** | 1.050 (1.032–1.068) | 1.048 (1.029–1.066) | 1.045 (1.026–1.064) | 1.015 (0.987–1.045) |
| **CO** | 1.028 (1.018–1.039) | 1.029 (1.018–1.040) | 1.025 (1.013–1.037) | 1.010 (0.992–1.029) |
| **$SO_2$** | 1.083 (1.068–1.099) | 1.087 (1.071–1.102) | 1.057 (1.041–1.073) | 1.108 (1.081–1.137) |
| **$O_3$** | 1.000 (0.988–1.013) | 1.000 (0.987–1.013) | 1.006 (0.993–1.019) | 0.997 (0.977–1.018) |
| **Mixture** | 1.072 (1.009–1.140) | 1.068 (1.003–1.138) | 1.102 (1.026–1.182) | 1.019 (0.933–1.114) |

Abbreviations: RR, relative risk; CI, confidence interval; $NO_2$, nitrogen dioxide; CO, carbon monoxide; $SO_2$, sulfur dioxide; $O_3$, ozone; CVD, cardiovascular disease; RD, respiratory disease; IQR, interquartile range.

**Table S3** Age-stratified associations between long-term exposure to gaseous air pollutants and mortality outcomes.

| Cause | Age | $NO_2$, per IQR rise | | CO, per IQR rise | | $SO_2$, per IQR rise | | $O_3$, per IQR rise | |
|---|---|---|---|---|---|---|---|---|---|
| | | RR (95% CI) | *P*-value | RR (95% CI) | *P*-value | RR (95% CI) | *P*-value | RR (95% CI) | *P*-value |
| Total mortality | | | | | | | | | |
| | ≤64 | 1.075 (1.054–1.096) *** | [Ref.] | 1.041 (1.029–1.053) *** | [Ref.] | 1.071 (1.054–1.088) *** | [Ref.] | 0.999 (0.985–1.013) | [Ref.] |
| | 65–74 | 1.069 (1.050–1.089) *** | 0.722 | 1.023 (1.011–1.034) *** | 0.030 | 1.078 (1.063–1.094) *** | 0.574 | 1.020 (1.008–1.033) ** | 0.023 |
| | ≥75 | 1.065 (1.045–1.085) *** | 0.505 | 1.024 (1.012–1.036) *** | 0.057 | 1.084 (1.068–1.101) *** | 0.280 | 1.002 (0.989–1.016) | 0.715 |
| Nonaccidental mortality | | | | | | | | | |
| | ≤64 | 1.067 (1.045–1.089) *** | [Ref.] | 1.044 (1.031–1.057) *** | [Ref.] | 1.086 (1.069–1.104) *** | [Ref.] | 0.994 (0.980–1.009) | [Ref.] |
| | 65–74 | 1.069 (1.050–1.088) *** | 0.883 | 1.023 (1.011–1.034) *** | 0.014 | 1.078 (1.062–1.093) *** | 0.484 | 1.021 (1.008–1.034) ** | 0.006 |
| | ≥75 | 1.064 (1.044–1.084) *** | 0.847 | 1.024 (1.012–1.036) *** | 0.023 | 1.082 (1.066–1.099) *** | 0.731 | 1.003 (0.989–1.016) | 0.408 |
| CVD mortality | | | | | | | | | |
| | ≤64 | 1.076 (1.051–1.101) *** | [Ref.] | 1.037 (1.022–1.052) *** | [Ref.] | 1.077 (1.057–1.098) *** | [Ref.] | 0.999 (0.983–1.015) | [Ref.] |
| | 65–74 | 1.077 (1.053–1.100) *** | 0.974 | 1.026 (1.012–1.041) *** | 0.315 | 1.048 (1.029–1.068) *** | 0.044 | 1.022 (1.007–1.038) ** | 0.039 |
| | ≥75 | 1.072 (1.051–1.093) *** | 0.795 | 1.024 (1.011–1.037) *** | 0.199 | 1.046 (1.029–1.064) *** | 0.023 | 1.010 (0.996–1.025) | 0.305 |
| RD mortality | | | | | | | | | |
| | ≤64 | 1.073 (1.027–1.122) ** | [Ref.] | 1.022 (0.992–1.053) | [Ref.] | 1.114 (1.071–1.159) *** | [Ref.] | 1.002 (0.969–1.035) | [Ref.] |
| | 65–74 | 1.076 (1.041–1.112) *** | 0.934 | 1.021 (0.997–1.044) | 0.930 | 1.072 (1.039–1.106) *** | 0.137 | 1.035 (1.009–1.061) ** | 0.124 |
| | ≥75 | 1.009 (0.980–1.040) | 0.024 | 1.005 (0.985–1.026) | 0.349 | 1.114 (1.084–1.144) *** | 0.999 | 0.989 (0.967–1.011) | 0.531 |

Abbreviations: ***P***-value <0.05 indicates statistically significant effect heterogeneity between subgroups; $NO_2$, nitrogen dioxide; CO, carbon monoxide; $SO_2$, sulfur dioxide; $O_3$, ozone; CVD, cardiovascular disease; RD, respiratory disease; RR, relative risk; CI, confidence interval; IQR, interquartile range.

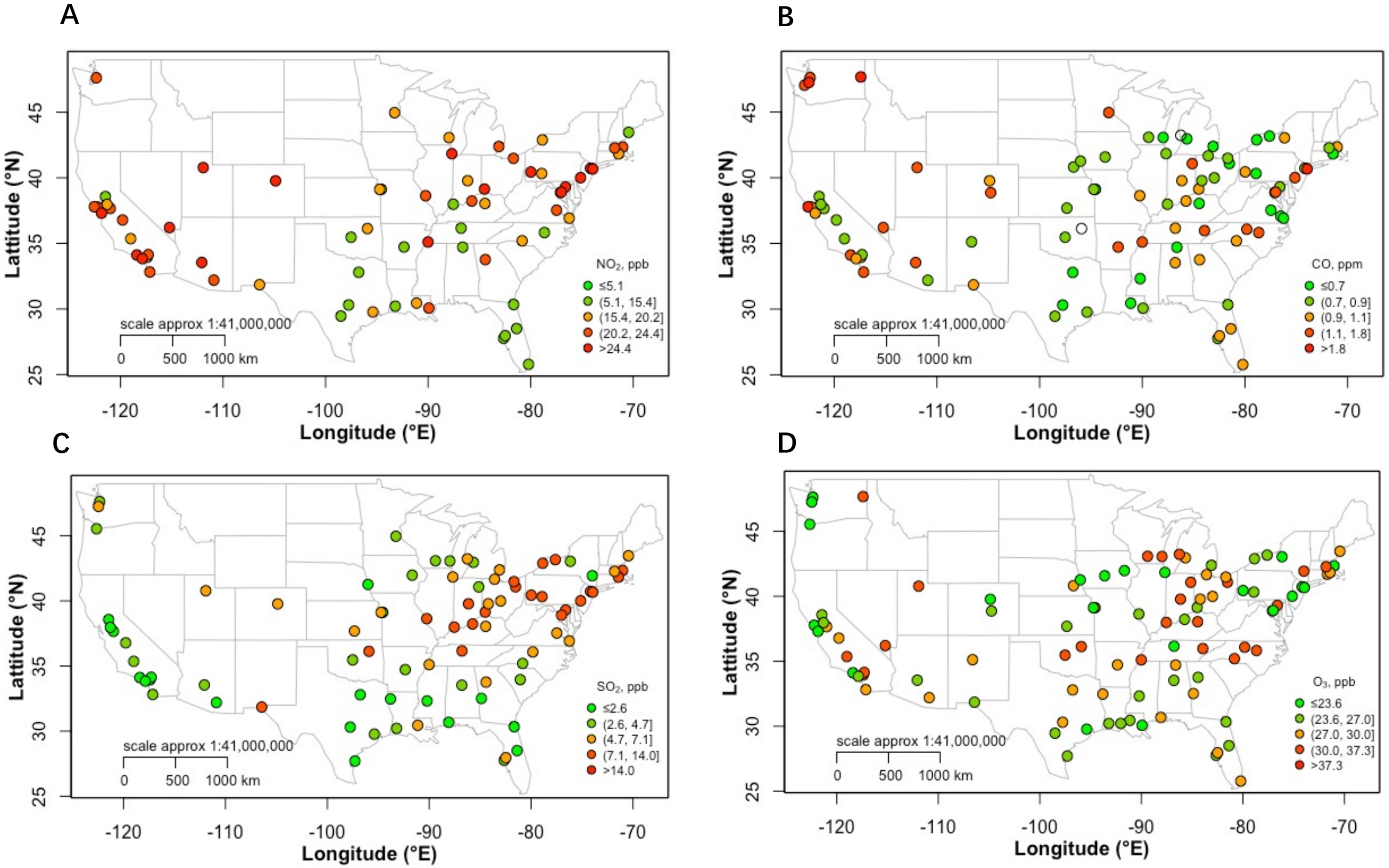


**Fig. S1** Annual average concentrations of specific gaseous pollutants ($NO_2$, CO, $SO_2$, $O_3$) in 108 US cities spanning the period 1987−2000. Abbreviations: $NO_2$, nitrogen dioxide; CO, carbon monoxide; $SO_2$, sulfur dioxide; $O_3$, ozone; ppb, part per billion; ppm, part per million.

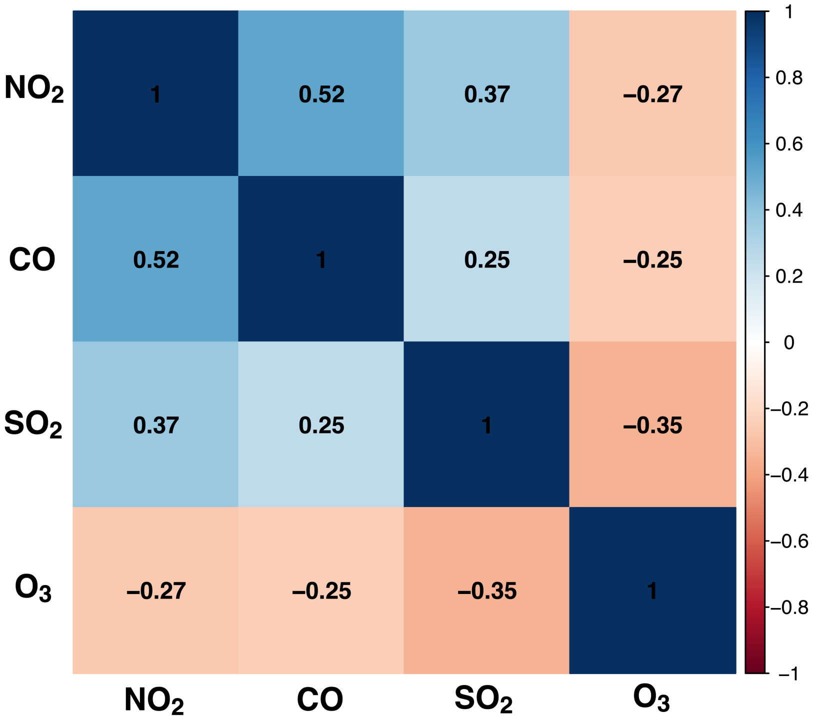


**Fig. S2** Spearman correlation coefficients between air pollutants in 108 US cities for the period 1987−2000. Abbreviations: $NO_2$, nitrogen dioxide; CO, carbon monoxide; $SO_2$, sulfur dioxide; $O_3$, ozone.
